# A three-year whole genome sequencing perspective of *Enterococcus faecium* sepsis in Australia

**DOI:** 10.1371/journal.pone.0228781

**Published:** 2020-02-14

**Authors:** Terence Lee, Stanley Pang, Marc Stegger, Shafi Sahibzada, Sam Abraham, Denise Daley, Geoffrey Coombs, on behalf of the Australian Group on Antimicrobial Resistance

**Affiliations:** 1 Antimicrobial Resistance and Infectious Diseases Research Laboratory, Murdoch University, Murdoch, Australia; 2 Statens Serum Institute, Copenhagen, Denmark; 3 Australian Group on Antimicrobial Resistance (AGAR), Fiona Stanley Hospital, Murdoch, Australia; Zhejiang University, CHINA

## Abstract

**Background:**

Over the last three decades, hospital adapted clonal complex (CC) 17 strains of *Enterococcus faecium* have acquired and exchanged antimicrobial resistance genes leading to the widespread resistance to clinically important antimicrobials globally. In Australia, a high prevalence of vancomycin resistance has been reported in *E*. *faecium* in the last decade.

**Methods:**

In this study, we determined the phylogenetic relationship and genetic characteristics of *E*. *faecium* collected from hospitalized patients with blood stream infections throughout Australia from 2015 to 2017 using high throughput molecular techniques.

**Results:**

Using single nucleotide polymorphism based phylogenetic inference, three distinct clusters of isolates were observed with additional sub-clustering. One cluster harboured mostly non-CC17 isolates while two clusters were dominant for the *vanA* and *vanB* operons.

**Conclusion:**

The gradual increase in dominance of the respective van operon was observed in both the vanA and vanB dominant clusters suggesting a strain-van operon affinity. The high prevalence of the van operon within isolates of a particular sub-cluster was linked to an increased number of isolates and 30-day all-cause mortality. Different dominant sub-clusters were observed in each region of Australia. Findings from this study can be used to put future surveillance data into a broader perspective including the detection of novel *E*. *faecium* strains in Australia as well as the dissemination and evolution of each strain.

## Introduction

*Enterococcus faecium* is a species of Gram-positive bacterium most commonly found as a commensal of the mammalian gut microbiome. However not all *E*. *faecium* strains exists solely as commensals, with some harbouring the ability to cause invasive infections [1]. In patients with bacteraemia, the vast majority (>90%) of enterococci identified were either *E*. *faecalis* or *E*. *faecium*. Although *E*. *faecalis* are more often responsible for infections, it is the nosocomial *E*. *faecium* infections that are typically resistant to multiple antimicrobial classes.

The *E*. *faecium* responsible for the majority of hospital acquired infections consists of various sequence types (STs) related to clonal complex (CC) 17 [2]. The acquisition of vancomycin resistance genes in CC17 is of serious concern as vancomycin is also used for the treatment of other Gram-positive bacterial infections including methicillin-resistant *Staphylococcus aureus* [3]. Of the known *van* genotypes conferring vancomycin resistance, the *vanA*, *vanB* and *vanM* genotypes have the greatest clinical significance as they confer intermediate to high levels of resistance and reside on genetic elements that can not only be transferred within enterococci but to other bacteria [4–6].

To ensure antimicrobial agents provide the best treatment outcomes with minimal risk of adverse effects, including the development of antimicrobial resistance [7], robust surveillance of antimicrobial resistant bacteria is required. In Australia, the Australian Group on Antimicrobial Resistance’s (AGAR) Australian Enterococcal Sepsis Outcome Program (AESOP) closely monitors enterococcal isolates from episodes of blood stream infection (BSI) across the country. In the 2015, 2016 and 2017 AESOPs the percentage of *E*. *faecium* BSIs that were vancomycin resistant was 46.5%, 50.1% and 47.0%, respectively [8–10]. Compared to the population-weighted mean of 8.3%, 11.8% and 14.2% for nations within the European Economic Area over the same time period [11–13], the high prevalence of vancomycin-resistant *E*. *faecium* (VREFm) BSIs in Australia represents a serious public health concern [14].

To understand the high prevalence of nosocomial VREfm causing enterococcal bacteraemia in Australia, a whole genome sequencing (WGS) bioinformatics approach was used to determine the relationship and characteristics of *E*. *faecium* isolates from the 2015, 2016 and 2017 AESOPs.

## Methods

Isolates were sourced from the 2015, 2016 and 2017 AGAR AESOPs. As part of the AESOPs, enterococcal isolates were referred to a central reference laboratory where WGS was performed on all *E*. *faecium* isolates. Libraries for WGS were prepared with the Illumina^®^ Nextera^®^ XT DNA Library Prep Kit (Illumina, United States) according to the prescribed protocol. Sequencing was performed on either the Miseq^™^ or NextSeq^™^ platform using the 600-cycle Reagent Kit v3 and the 300-cycle mid output Reagent Kits v2 respectively. As part of quality control, sequence data of isolates yielding less than 40x depth and 50% coverage to the reference chromosome of *E*. *faecium* Aus0004 (GenBank CP003351), were excluded from the study.

For each isolate, raw sequencing reads were cleaned using Trimmomatic V0.38 [15] before being assembled by SPAdes V3.12.0 [16] followed by gene identification and annotation by Prokka V1.13 [17]. Pan genome analysis was performed using Roary V3.12.0 [18] on Prokka generated files. Virulence and resistance genes were identified using ABRicate V0.8.7 in tandem with VFDB (21/01/2019) [19] and the ResFinder (21/01/2019) [20] databases respectively. Positive identification of resistance genes and virulence factors were indicated by a minimum sequence homology of 85% to respective database entries. Analysis of the *esp* virulence factor was excluded from this study due to the limitations of the sequencing methodology in resolving repeat regions within the *esp* gene [21]. Functional van operons were determined by the presence of regulation genes (*vanR* and *vanS*) and essential genes (*vanH*, *vanA*/*vanB*, vanX) within the respective vanA or vanB operon. The ST for each isolate was determined using the scheme described by Homan *et al*. [22] with sequence definitions hosted on the BIGSdb (https://pubmlst.org/software/database/bigsdb/) database [23]. Isolates with one or more ambiguous MLST alleles were categorized as undefined and excluded from the study. The BURST diagram describing the relationship of STs was constructed using eBURST V3 [24]. Single nucleotide polymorphisms (SNPs) were identified using Snippy V4.1.0 [25]. Removal of recombinant DNA segments was performed on SNP alignments using ClonalFrameML V1.11 [26]. Phylogenetic trees were constructed using the maximum likelihood method based on the SNP alignments in RAxML V8.2.11 [27] with the GTRCAT model and bootstrap values set at 1000. The main phylogenetic tree was constructed using all isolates with the root placed the most distantly related isolate. The most distantly related isolate was determined by SNP inferred phylogeny of isolates with a distantly related reference from the *E*. *durans* species (Accession: CP012366). Clusters were determined based on distinct phylogenetic branches observed. To enhance SNP resolution in major branches of the main phylogenetic tree, SNP analysis was repeated on subsets of isolates labelled as clusters. Individual cluster phylogeny was analysed using the previously described method with the root placed at the most distantly related isolate determined by the main phylogenetic tree. Further sub-clustering was performed based on distinct branches of each phylogenetic tree. The iTOL V3 [28] web service was used to visualize the phylogenetic tree along with the metadata.

All statistical analysis were performed with the statistical package R [29]. The association between clusters and mortality were investigated using generalised linear regression model (GLM). Odds ratios and confidence intervals were calculated from the GLM model with p-values ≤ 0.05 considered significant.

### No ethics approval was required

Nucleotide sequences of isolates from AGAR AESOP 2015, 2016 and 2017 have been deposited in the sequence read archive under BioProject IDs: PRJNA562395, PRJNA562414 and PRJNA5662407 respectively.

## Results

From 1^st^ January 2015 to 31^st^ December 2017 1,296 *E*. *faecium* BSI isolates were collected in the AGAR AESOPs. WGS quality control excluded 266 isolates and an additional five isolates were excluded due to ambiguous MLST alleles (two isolates with multiple copies of an allele and three isolates with truncated alleles). The remaining 1,025 isolates (332 in 2015, 320 in 2016 and 373 in 2017) were used in the study. The average genome size of isolates was 2,974,436bp with a standard deviation of 269,356bp. The average number of genes per isolate was 2,789 with a standard deviation of 244.

For the 1,025 episodes of *E*. *faecium* bacteraemia, the average patients age was 64 years with an interquartile range of 55 and 77. The male to female ratio was approximately 1.6 and when known, the 30-day all-cause mortality was 26.2%.

*E*. *faecium* isolates were collected from all Australian states and mainland territories: New South Wales (NSW) 352 isolates; Victoria (Vic) 301; Western Australia (WA) 140; Queensland (Qld) 85; South Australia (SA) 77; Tasmania (Tas) 34; Australian Capital Territory (ACT) 21; and the Northern Territory (NT) 15.

Of the 1,025 isolates, 979 were categorized into 75 previously reported STs. The remaining 46 isolates consisted of 43 new MLST profiles which were designated a ST by the MLST database curator (https://pubmlst.org/efaecium/). From the BURST model (S1 Fig), 95.8% of isolates (982/1025), which included 83 STs were grouped into CC17. Thirty-two STs (40 isolates) were grouped into CC94 with ST94 identified as the founder. The three remaining isolates were singletons and a doubleton. Overall, 11 STs (ST17, ST18, ST78, ST80, ST192, ST203, ST262, ST555, ST796, ST1421 and ST1424) were considered as major STs (≥ 20 isolates) and all belonged to CC17 (Table 1).

**Table 1:** Eleven major sequence types (≥20 isolates) identified in the Australian Group on antimicrobial resistance Australian Enterococcal Sepsis Outcome Program 2015–2017.

	ST17	ST18	ST78	ST80	ST192	ST203	ST262	ST555	ST796	ST1421	ST1424
2015	17	5	21	28	9	33	9	43	66	45	5
2016	40	4	11	40	5	20	9	31	47	52	16
2017	60	11	10	35	7	10	3	17	46	52	50
Total	117	20	42	103	21	63	21	91	159	149	71

Of the 11 major STs, ST1421 and ST796 had the most number of isolates with 149 and 159 isolates respectively. Over the three years, the number of isolates identified as ST17 and ST1424 increased the most per year from 17 to 60 and 5 to 45 isolates respectively. Conversely, the number of isolates identified as ST555 and ST203 decreased the most per year from 43 to 17 and 33 to 10 isolates respectively.

From the BURST analysis, the recently reported *pstS*-absent STs [30] were identified in three different branches within CC17 and were separated by at least three ST nodes. The largest group of *pstS*-absent STs consisted of ST1421 as the founder with ST1422, ST1423, ST1478, ST1553 and ST1558 as single locus variants (SLVs). ST1424 and ST1559 formed the second group while the third group consisted of a single ST1545 isolate.

Sixty-one virulence factors were identified, of which nine were present in five or more isolates. The nine predominant virulence factors identified were: *cpsF* (encodes cleavage and polyadenylation specific factor); *ecbA* (encodes a collagen type-V binding microbial surface component recognizing adhesive matrix molecule [MSCRAMM]); *fss3* (encodes a fibrinogen-binding MSCRAMM); *psaA* (encodes pneumococcal surface adhesion A); *sgrA* (encodes a LPxTG surface adhesion that binds to fibrinogen and nidogen and is commonly implicated in biofilm formation); *acm* (encodes a collagen binding MSCRAMM); *bsh* (encodes bile salt hydrolase), *bopD* (putatively encodes a sugar-binding transcriptional regulator critical for the process of biofilm); and *clpP* (encodes the ClpP protease) were. The average number of predominant virulence factors identified per isolate was 5.2. The percentage of isolates harbouring the nine predominant virulence factors for the eleven major STs is summarized in S1 Table.

Twenty-six antimicrobial resistance genes were identified of which 20 were present in five or more isolates. The 20 predominant antimicrobial resistance genes identified were: *aadE*, *aac(6')-aph(2'')*, *ant(6)-Ia*, *aph(2'')-Ie*,*aph(3')-III* and *spc* encoding aminoglycoside resistance; *cat*(pC221) and *cat* encoding chloramphenicol resistance; *tet*(S), *tet*(L), *tet*(M), *tet*(U) encoding tetracycline resistance; *erm*(A), *erm*(B) *and erm*(T) encoding macrolide, lincosamide and streptogramin B resistance; *vanA* and *vanB* encoding glycopeptide resistance; *lnu*(B) encoding lincosamide resistance; *dfrG* encoding trimethoprim resistance; and *msr*C encoding erythromycin, macrolide and streptogramin B resistance. The average number of predominant resistance genes identified per isolate was 6.7. The percentage of isolates harbouring the 20 predominant antimicrobial resistance genes for the 11 major STs is summarized in S2 Table. Vancomycin resistance in isolates from all three years was limited to the vanA and vanB operon. Neither vanM nor the vertically transmitted vanC operons were identified. The proportion of *E*. *faecium* isolates harbouring the vanA operon increased throughout the study period from 17.2% in 2015 to 22.3% in 2017 while the proportion of isolates harbouring the vanB operon decreased from 38.3% in 2015 to 25.2% in 2017. The geographical distribution of the proportions of van operon was different for each state and mainland territory (Table 2).

**Table 2:** The percentage of *van* genotypes across Australian states and mainland territories.

	ACT	NSW	NT	Qld	SA	Tas	Vic	WA
*vanA*	47.6	38.6	0.0	10.6	3.9	2.9	12.6	6.4
*vanB*	4.8	13.1	73.3	32.9	46.8	32.4	53.8	8.6
*van* negative	47.6	48.3	26.7	56.5	49.4	64.7	33.6	85.0

**Fig 1:**
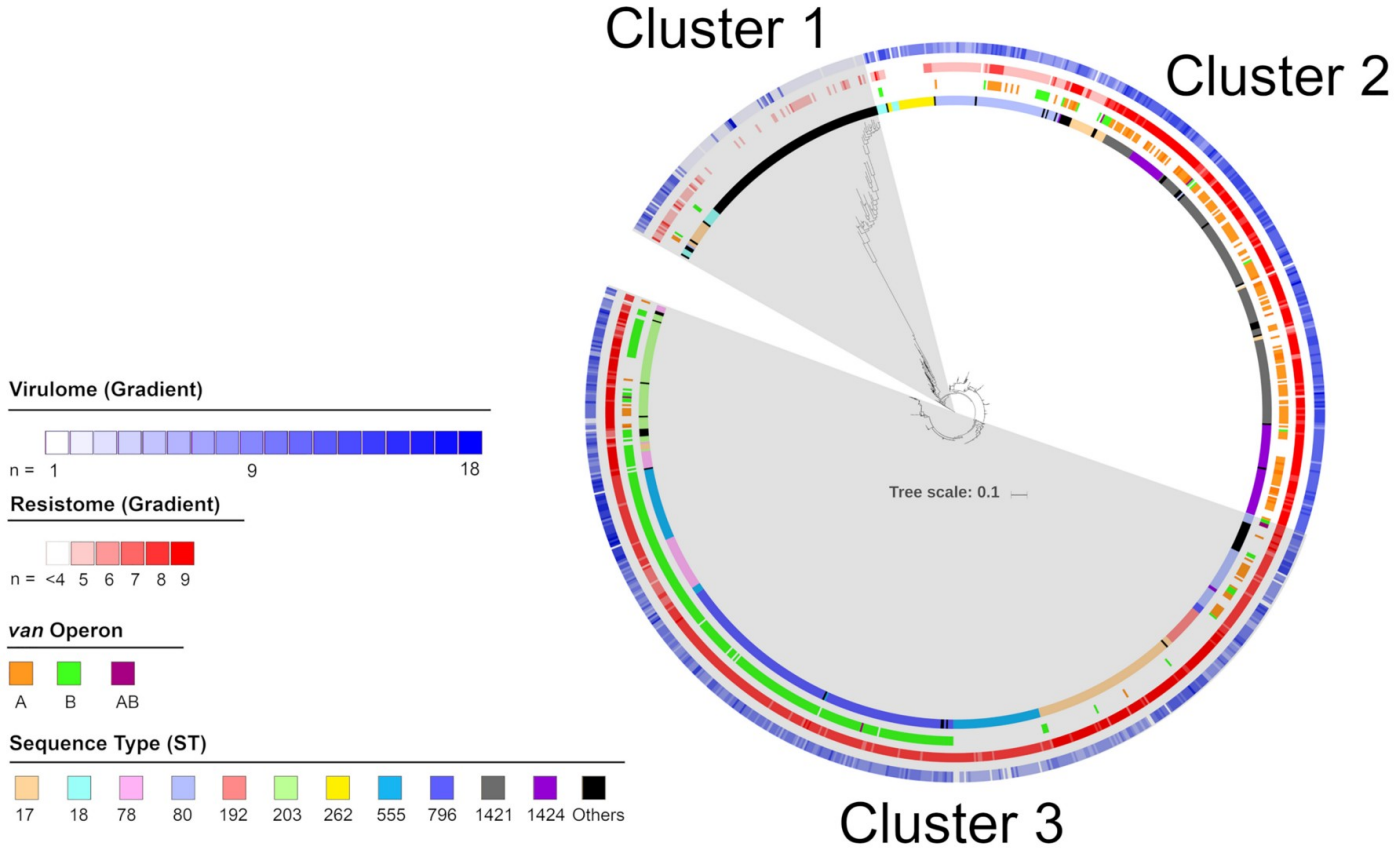
Maximum likelihood phylogenetic tree based on single nucleotide polymorphisms grouped all *Enterococcus faecium* isolates into three clusters. From the outermost ring: Number of virulence factors, number of antimicrobial resistance genes not including the van operon, type of van operon and major sequence types are highlighted as per legend.

A maximum likelihood phylogenetic tree was constructed based on 35,058 core single nucleotide polymorphisms (SNPs) to determine the relatedness between isolates (Fig 1). The root of the tree was placed at the most distantly related isolate (E31901_2017). Three major clusters were observed. From the phylogenetic tree, the major STs 78, 192, 203, 262, 555, 796, 1421 appeared in one cluster; while STs, 17, 18, 80 and 1424 appeared in more than one cluster (Table 3).

**Table 3:** Isolate characteristics based on the three phylogenetic clusters.

Cluster	Sub-cluster	n	Major STs^1^	30-day all-cause mortality^2^ (%)	Mortality odds ratio (95% CI)	Average antimicrobial resistance genes (n)	Average virulence factors (n)
1	A	30	ST17, ST18 and ST80	23.1	1.9 (0.6–5.7)	4.8	5.2
	B	104	-	13.6	ref	1.6	4.0
2	A	54	ST17, ST18 and ST262	24.5	2.1 (0.8–5.2)	4.7	5.2
	B	85	ST80, ST1421 and ST1424	32.5	3.1 (1.4–7.0)	5.3	5.7
	C	222	ST17, ST1421 and ST1424	33.0	3.1 (1.6–6.6)	6.9	7.7
3	A	73	ST17, ST80, ST192, ST203 and ST1424	24.3	2.0 (0.9–4.8)	5.2	7.5
	B	69	ST17, ST80 and ST192	23.9	2.0 (0.9–4.8)	4.0	7.7
	C	71	ST203	17.2	1.3 (0.5–3.3)	5.7	7.4
	D	18	ST18	43.8	4.9 (1.5–16.2)	5.8	6.8
	E	86	ST17 and ST555	25.3	2.2 (1.0–5.0)	5.4	6.9
	F	214	ST78, ST80, ST203, ST555 and ST796	26.6	2.3 (1.2–4.9)	5.6	6.9

^1^ STs with 20 or more isolates

^2^ where the patient 30-day all-cause mortality was known

**Fig 2:**
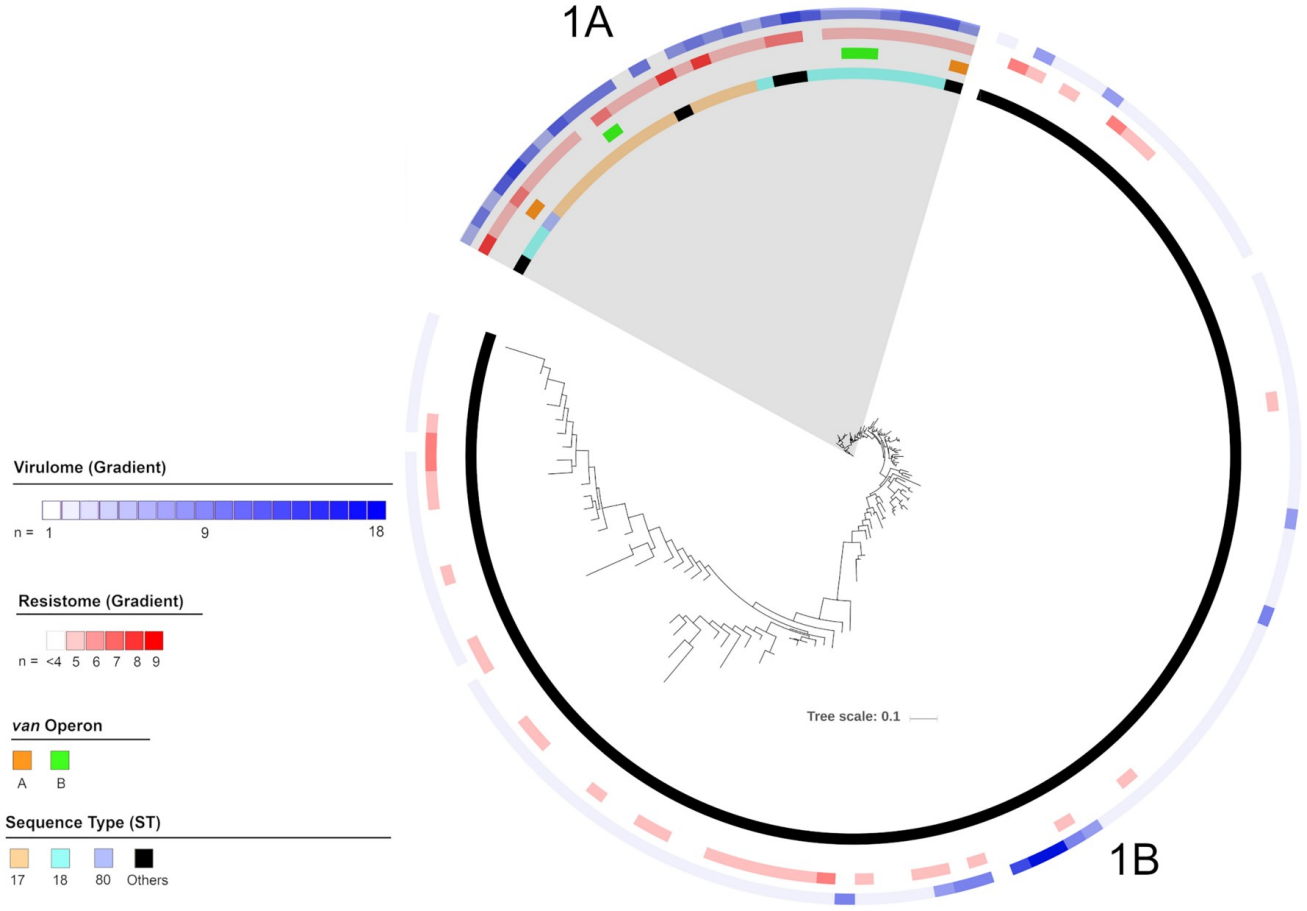
Maximum likelihood phylogenetic tree of cluster 1 isolates. From the outermost ring: Number of virulence factors, number of resistance genes not including the van operon, type of van operon and sequence types are highlighted as per legend.

Of the three clusters, cluster 1 harboured the fewest isolates, having only 134 isolates (Fig 2). However in terms of STs, cluster 1 was the most diverse with 84 unique STs including the major STs 17, 18 and 80. Cluster 1 isolates could be divided into two sub-clusters: 1A and 1B. Sub-cluster 1A consisted of 30 isolates with eight STs, all members of CC17, and included the major STs 17, 18 and 80. Sub-cluster 1B consisted of 104 isolates with 76 STs and included all CC94 isolates, which accounted for 38.5% of isolates in the sub-cluster and, the three non-CC94/CC17 isolates. No isolates from the major STs were identified in sub-cluster 1B.

Isolates from sub-cluster 1A harboured an average of 4.8 antimicrobial resistance genes. All isolates in sub-cluster 1A harboured *msrC* and more than 80% of isolates harboured *tet*(L) and *dfrG*. No isolate in sub-cluster 1A harboured the *aadE*, *cat*(pC221) or *tet*(S) antimicrobial resistance genes. The vanA and vanB operon was identified in 6.7% and 10.0% of isolates respectively. No isolate harboured both van operons. Sub-cluster 1A isolates harboured an average of 5.2 virulence factors. All isolates in sub-cluster 1A harboured the *acm*, *bsh* and *clpP* and more than 80% harboured *bopD* and *sgrA*. No isolates in sub-cluster 1A harboured the *cpsF* virulence factor. When known, the 30-day all-cause mortality of patients with sub-cluster 1A isolates was 23.1%. When compared to sub-cluster 1B, which had the lowest mortality of all sub-clusters at 13.6%, the odds ratio for mortality was calculated at 1.9 (CI: 0.6–5.7). The difference in 30-day all-cause mortality between sub-clusters 1A and 1B was not significant (p = 0.25).

Isolates from sub-cluster 1B harboured an average of 1.6 antimicrobial resistance genes. More than 80% of isolates harboured *msrC*. No isolates in sub-cluster 1B harboured the *vanA*, *vanB*, *ant(6)-la*, *aph(2”)-le* and *tet*(S) resistance genes. Sub-cluster 1B isolates harboured an average of 4.0 virulence factors. All isolates harboured *bopD* and *clpP*, and more than 80% harboured *bsh*. None of the isolates in sub-cluster 1B harboured the *psaA* virulence factor.

**Fig 3:**
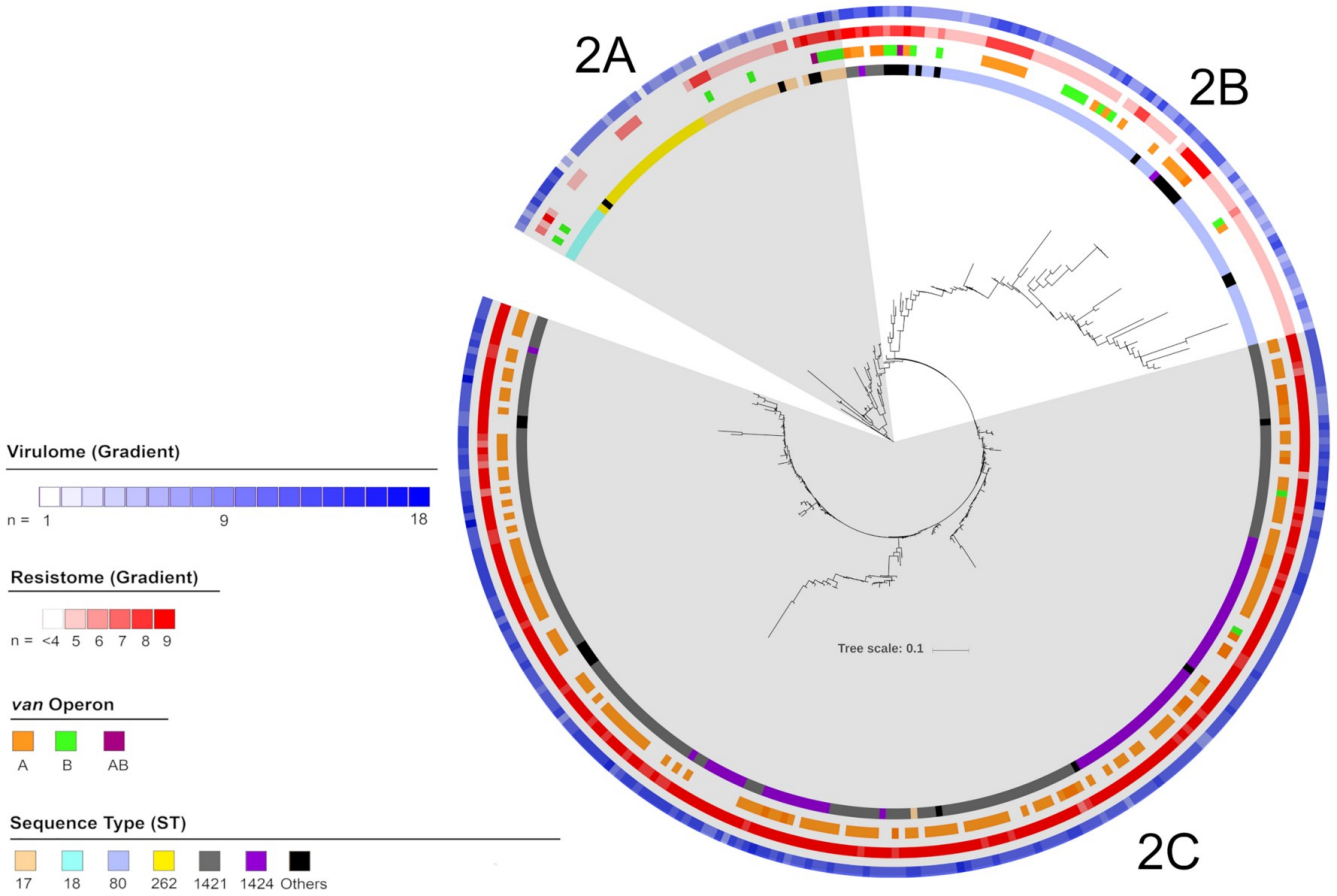
Maximum likelihood phylogenetic tree of cluster 2 isolates. From the outermost ring: Number of virulence factors, number of resistance genes not including the van operon, type of van operon and sequence types are highlighted as per legend.

Cluster 2 consisted of 361 isolates with 22 STs, all part of CC17, and included five major STs: 18, 262, 80, 1424, 1421 (Fig 3). Cluster 2 isolates could be divided into three sub-clusters: 2A, 2B and 2C. Sub-cluster 2A, consisted of 54 isolates with six STs including the major STs 17, 18 and 262. Isolates from sub-cluster 2A were collected from all Australian regions except the ACT. Sub-cluster 2B, consisted of 85 isolates with 12 STs including major STs 1421, 1424 and 80. Isolates from sub-cluster 2B were collected from across Australia. Sub-cluster 2C, consisted of 222 isolates with eight STs including major STs 17, 1421 and 1424. Isolates from sub-cluster 2C were only collected from NSW, Vic, SA and the ACT.

Isolates from sub-cluster 2A harboured an average of 4.7 antimicrobial resistance genes. All isolates in sub-cluster 2A harboured *msrC* and more than 80% of isolates harboured *dfrG*. No isolate in sub-cluster 1A harboured *aph(2”)-le*, *erm*(A), *tet*(S) or *spc*. The vanA and vanB operon was identified in 1.9% and 16.7% of isolates respectively. Sub-cluster 2A isolates harboured an average of 5.2 virulence factors. All isolates in sub-cluster 2A harboured *acm*, *bopD*, *bsh* and *clpP*. None of the remaining predominant virulence factors were identified in more than 80% of isolates. No isolate in sub-cluster 2A harboured *cpsF*. When known, the 30-day all-cause mortality of patients with 2A isolates was 24.5%. Compared to sub-cluster 1B isolates the odds ratio for mortality was calculated at 2.1(CI: 0.5–5.2). The difference in 30-day all-cause mortality between sub-clusters 2A and 1B was not significant (p = 0.12).

Isolates from sub-cluster 2B harboured an average of 5.3 antimicrobial resistance genes. All isolates in sub-cluster 2B harboured *msrC* and more than 80% of isolates harboured *dfrG*. No isolates in sub-cluster 2B harboured *aph(2”)-le* or *cat*. The vanA and vanB operon was identified in 28.2% and 14.1% of isolates respectively. Sub-cluster 2B isolates harboured an average of 5.7 virulence factors. All isolates in sub-cluster 2B harboured *bopD* and *clpP* and more than 80% of isolates harboured *acm*, *bsh* and *sgrA*. No isolates in sub-cluster 2B harboured the *cpsF* virulence factor. When known, the 30-day all-cause mortality of patients with sub-cluster 2B isolates was 32.5%. Compared to sub-cluster 1B isolates the odds ratio for mortality was calculated at 3.1 (CI: 1.4–7.0). The difference in 30-day all-cause mortality between sub-clusters 2B and 1B was significant (p = 0.005).

Isolates from sub-cluster 2C harboured an average of 6.9 antimicrobial resistance genes. More than 80% of isolates harboured *dfrG*, *erm*(A), *erm*(B), *msr*C, *spc* and *aph(3’)-III*. No isolates in sub-cluster 2C harboured *aadE*, *ant(6)-la*, *aph(2”)-le*, *cat*(p221), *cat*, *lnu*(B) or *tet*(S). The vanA and vanB operon was identified in 68.0% and 0.9% of isolates respectively. Sub-cluster 2C isolates harboured an average of 7.7 virulence factors. All isolates in sub-cluster 2C harboured *acm*, *bopD* and *clpP* and more than 80% of isolates harboured *bsh*, *ecbA*, *fss3*, *psaA* and *sgrA*. No isolates in sub-cluster 2A harboured the *cpsF* virulence factor. When known, the 30-day all-cause mortality of patients with sub-cluster 2C isolates was 33.0%. Compared to sub-cluster 1B isolates the odds ratio for mortality was calculated at 3.1 (CI: 1.6–6.6). The difference in 30-day all-cause mortality between sub-clusters 2C and 1B was significant (p = 0.001).

**Fig 4:**
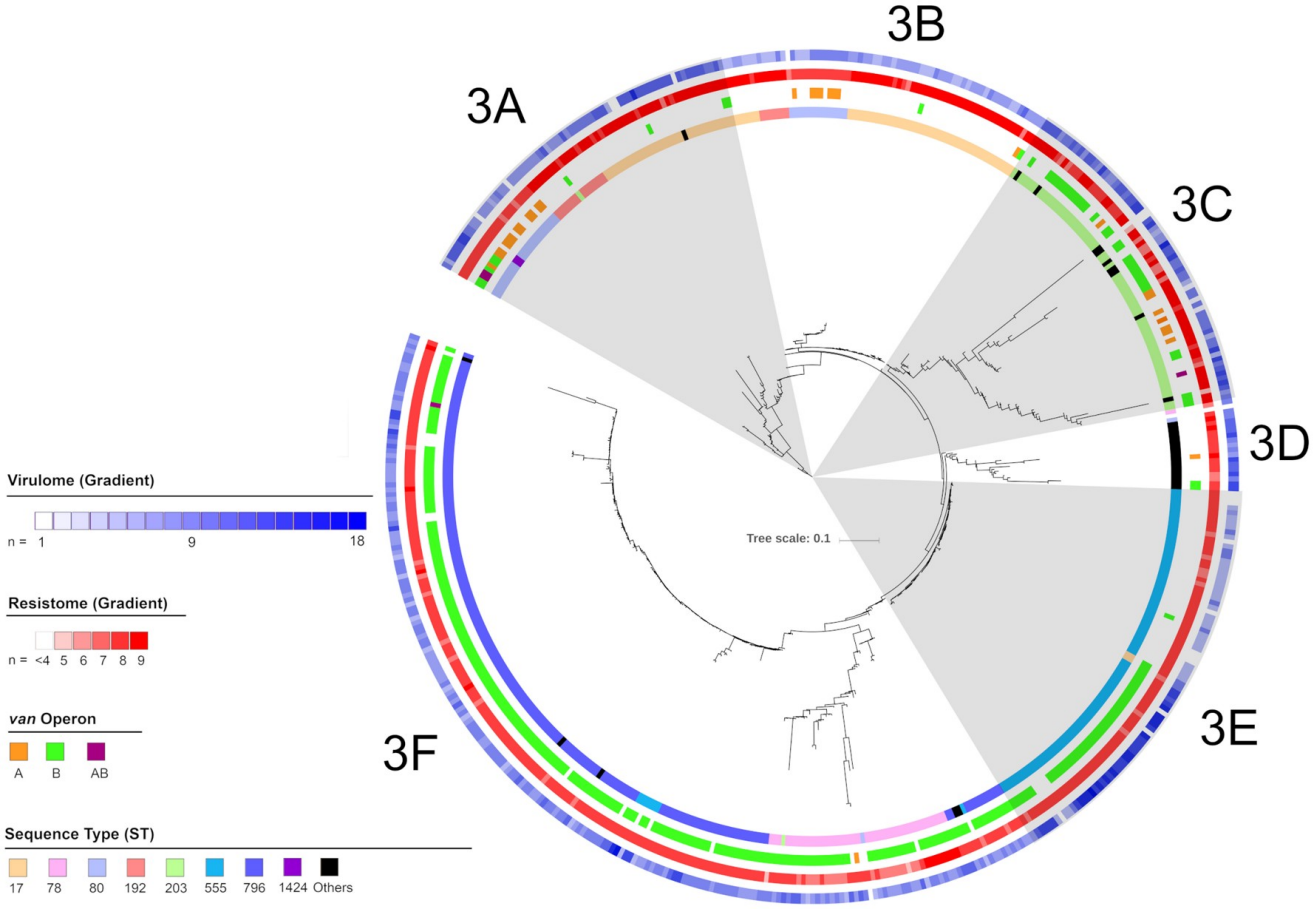
Maximum likelihood phylogenetic tree of isolates in cluster 3. From the outermost ring: Number of virulence factors, number of resistance genes not including the van operon, type of van operon and sequence types are highlighted as per legend.

Cluster three was the largest cluster consisting of 530 isolates with 20 STs (Fig 4). All STs identified in cluster 3 were part of CC17 and included the major STs 17, 78, 80, 203, 555, 796, 1424, 1421. Cluster 3 isolates could be divided into six sub-clusters: 3A to 3F. Sub-cluster 3A, consisted of 73 isolates with six STs including the major STs 17, 80, 192, 203 and 1424. Isolates from sub-cluster 3A were collected from all Australian regions except SA, ACT and NT. Sub-cluster 3B, consisted of 69 isolates with three STs including the major STs 17, 80 and 192. Isolates from sub-cluster 3B were collected from all Australian regions except NSW, Vic and ACT. Sub-cluster 3C, consisted of 71 isolates with seven STs including the major ST ST203. Isolates from sub-cluster 3C were collected from all Australian regions except the NT. Sub-cluster 3D, consisted of 18 isolates with four STs including the major STs 18 and 80. Isolates from sub-cluster 3D were collected from all Australian regions except the NT and ACT. Sub-cluster 3E, consisted of 86 isolates, with two major STs 17 and 555. Isolates from sub-cluster 3E were collected from all Australian regions except the ACT, Qld and Vic. Sub-cluster 3F, consisted of 214 isolates, with eight STs including the major STs 78, 80, 203, 555 and 796. Isolates from sub-cluster 3F were collected from all Australian regions except the ACT.

Isolates from sub-cluster 3A harboured an average of 5.2 antimicrobial resistance genes. All isolates in sub-cluster 3A harboured *msrC* and more than 80% of isolates harboured ant(6)-IA, *aph(3”)-III* and *erm*(B). No isolates in sub-cluster 3A harboured *aadE*, *aph(2”)-le*, *cat*(p221), *cat*, *erm*(A) or *spc*. The vanA and vanB operon was identified in 19.2% and 15.1% of isolates respectively. Sub-cluster 3A isolates harboured an average of 7.5 virulence factors. All isolates in sub-cluster 3A harboured *acm*, *bopD* and *fss3* and more than 80% of isolates harboured *bsh*, clpP, *psaA* and *sgrA*. No isolates in sub-cluster 3A harboured the *cpsF* virulence factor. When known, the 30-day all-cause mortality of patients with sub-cluster 3A isolates was 24.3%. Compared to sub-cluster 1B isolates the odds ratio for mortality was calculated at 2.0 (CI: 0.9–4.8). The difference in 30-day all-cause mortality between sub-clusters 3A and 1B was not significant (p = 0.10).

Isolates from sub-cluster 3B harboured an average of 4.0 antimicrobial resistance genes. All isolates in sub-cluster 3B harboured *msrC* and more than 80% of isolates harboured *aph(3’)-III* and *erm*(B). No isolate in sub-cluster 3B harboured *aadE*, *aph(2”)-Ie*, *cat(p221)*, *cat*, *erm*(A), *erm*(T), *spc* or *tet*(L). The vanA and vanB operon was identified in 11.2% and 2.9% of isolates respectively. Sub-cluster 3B isolates harboured an average of 7.7 virulence factors. All isolates in sub-cluster 3B harboured *acm*, *bopD* and *clpP* and more than 80% of isolates harboured *bsh*, *fss3*, *psaA* and *sgrA*. No isolates in sub-cluster 3B harboured the *cpsF* virulence factor. When known, the 30-day all-cause mortality of patients with sub-cluster 3B isolates was 23.9%. Compared to sub-cluster 1B isolates the odds ratio for mortality was calculated at 2.0 (CI: 0.9–4.8). The difference in 30-day all-cause mortality between sub-clusters 3B and 1B was not significant (p = 0.11).

Isolates from sub-cluster 3C harboured an average of 5.7 antimicrobial resistance genes. All isolates in sub-cluster 3C harboured *msrC* and more than 80% of isolates harboured *erm*(B) and *tet*(M). No isolates in sub-cluster 3C harboured *aadE*, *aph(2”)-le*, *cat*, *erm*(A), *erm*(T), *spc*, *tet*(L) or *tet*(S). The vanA and vanB operon was identified in 12.9% and 47.1% of isolates respectively. Sub-cluster 3C isolates harboured an average of 7.4 virulence factors. All isolates in sub-cluster 3C harboured *acm*, *bsh* and *clpP* and more than 80% of isolates harboured *bopD*, *fss3*, *psaA* and *sgrA*. No isolate in sub-cluster 3B harboured the *cpsF* virulence factor. When known, the 30-day all-cause mortality of patients with sub-cluster 3C isolates was 17.2%. Compared to sub-cluster 1B isolates the odds ratio for mortality was calculated at 1.3 (CI: 0.5–3.3). The difference in 30-day all-cause mortality between sub-clusters 3C and 1B was not significant (p = 0.55).

Isolates from sub-cluster 3D harboured an average of 5.8 antimicrobial resistance genes. All isolates in sub-cluster 3D harboured *msrC* and more than 80% of isolates harboured *ant(6)-Ia*, *aph(3”)-III* and *erm*(B). No isolates in sub-cluster 3D harboured *aadE*, *aph(2”)-Ie*, *cat(pC221)*, *erm*(A), *lnu*(B), *spc or tet*(S). The vanA and vanB operon was identified in 5.6% and 11.1% of isolates respectively. Sub-cluster 3D isolates harboured an average of 6.8 virulence factors. All isolates in sub-cluster 3D harboured *acm*, *bopD* and *clpP* and more than 80% of isolates harboured *bsh*, *psaA* and *sgrA*. No isolates in sub-cluster 3D harboured the *cpsF* virulence factor. When known, the 30-day all-cause mortality of patients with 3D isolates was 43.8%. Compared to sub-cluster 1B isolates the odds ratio for mortality was calculated at 4.9 (CI: 1.5–16.2). The difference in 30-day all-cause mortality between sub-clusters 3D and 1B was significant (p = 0.008).

Isolates from sub-cluster 3E harboured an average of 5.4 antimicrobial resistance genes. All isolates in sub-cluster 3E harboured *msr*C and more than 80% of isolates harboured *dfrG* and *erm*(B). No isolates in sub-cluster 3E harboured *aadE*, *aph(2”)-Ie*, *cat(pC221)*, *cat*, *vanA or tet*(S). The vanB operon was identified in 48.8% of isolates. The vanA operon was not identified in sub-cluster 3E. Sub-cluster 3E isolates harboured an average of 6.9 virulence factors. All isolates in sub-cluster 3E harboured *bopD*, *bsh*, *clpP* and *fss3* and more than 80% of isolates harboured *acm*, *psaA* and *sgrA*. No isolates in sub-cluster 3E harboured the *cpsF* or *ecbA* virulence factors. When known, the 30-day all-cause mortality of patients with sub-cluster 3E isolates was 25.3%. Compared to sub-cluster 1B isolates the odds ratio for mortality was calculated at 2.2(CI: 1.0–5.0). The difference in 30-day all-cause mortality between sub-clusters 3E and 1B was not significant (p = 0.06).

Isolates from sub-cluster 3F harboured an average of 5.6 antimicrobial resistance genes. All isolates in sub-cluster 3F harboured *msr*C and more than 80% of isolates harboured *dfrG*, *erm*(B) and *vanB*. No isolates in sub-cluster 3F harboured the *aadE* resistance gene. The vanA and vanB operon was identified in 0.9 and 92.1% of isolates respectively. Sub-cluster 3F isolates harboured an average of 6.9 virulence factors. All isolates in sub-cluster 3F harboured *bsh* and *clpP* and more than 80% of isolates harboured *acm*, *bopD*, *fss3*, *psaA* and *sgrA*. When known, the 30-day all-cause mortality of patients with sub-cluster 3F isolates was 26.6%. Compared to sub-cluster 1B isolates the odds ratio for mortality was calculated at 2.3(CI: 1.2–4.9). The difference in 30-day all-cause mortality between sub-clusters 3F and 1B was significant (p = 0.02).

Geographically, the distribution of van genes and sequence types varied. In NSW, 38.6% and 13.1% of isolates harboured the vanA and vanB operon respectively. An additional isolate harboured both vanA and vanB operons. The most frequent ST amongst the NSW isolates was ST1421 accounting for 32.4% of isolates. Overall, 54% of isolates from NSW were identified in sub-cluster 2C.

In Vic, 12.6% and 53.8% of isolates harboured the vanA and vanB operon respectively. An additional five isolates harboured both the vanA and vanB operons. Except for a pair of closely related ST80 isolates, isolates harbouring both operons were from different STs and unrelated phylogenetically. The most frequent ST amongst the Vic isolates was ST796 accounting for 44.5% of isolates. Overall, 47.2% of isolates from Vic were identified in sub-cluster 3D.

In Qld, 10.6% and 32.9% of isolates harboured the vanA and vanB operon respectively. The most frequent ST amongst the Qld isolates was ST17 accounting for 29.4% of isolates. Overall, 23.5% and 22.4% of isolates from Qld were identified in sub-cluster 3A and 3F respectively.

In WA, only 6.4% and 8.6% of isolates harboured the vanA and vanB operon respectively. The most frequent ST amongst the WA isolates was ST17 accounting for 35% of isolates. Overall, 40.0% of isolates from WA were identified in sub-cluster 3B.

In SA, 3.9% and 46.8% of isolates harboured the vanA and vanB operon respectively. The most frequent ST amongst the SA isolates was ST555 accounting for 45.5% of isolates. Overall, 45.5% of isolates from SA were identified in sub-cluster 3E.

In Tas, 2.9% and 32.4% of isolates harboured the vanA and vanB operon respectively. The most frequent ST amongst the Tas isolates was ST796 accounting for 29.4% of isolates. Overall, 23.5% of isolates from Tas were identified in sub-cluster 3B.

In the NT, 73.3% of isolates harboured the vanB operon. The most frequent ST amongst the NT isolates was ST555 accounting for 66.7% of isolates. Overall, 66.7% of isolates from NT were identified in sub-cluster 3E.

In the ACT, 47.6% and 4.8% of isolates harboured the vanA and vanB operon respectively. The most frequent ST amongst the ACT isolates was ST1421 accounting for 57.1% of isolates. Overall, 61.9% of isolates from ACT were identified in sub-cluster 2C.

## Discussion

In 2011, 36.5% of Australian *E*. *faecium* bacteraemia isolates were identified as vancomycin non-susceptible, of which 98.4% harboured the vanB operon [31]. Supported by AESOP reports, the high prevalence of VREfm has been a growing trend in Australian hospitals over the last decade. In this study, we employed WGS as a screening tool in the surveillance of 1,025 BSI associated *E*. *faecium* collected across Australia from 2015 to 2017. Using bioinformatics, we have demonstrated *E*. *faecium* epidemiology is heterogenous across Australia with a mixture of strains harbouring unique genetic compositions that are constantly evolving in each region. When studied on a national level, Australian *E*. *faecium* BSI isolates can be classified into three broad molecular clusters and further divided into eleven sub-clusters. In addition, by pairing genomic features with molecular phylogeny we have identified key phylogenetic clusters with increased clinical significance with respect to 30-day all-cause mortality.

Focusing on the high prevalence of VREfm in Australia, the majority of vancomycin resistant isolates were associated with sub-clusters 2 and 3. Within clusters 2 and 3, sub-clusters 2C and 3F had the highest proportion of vancomycin resistant isolates. Aside from the high van operon carriage, sub-cluster 2C had the most number of isolates, the highest number of average antimicrobial resistance and virulence genes per isolate, and the second highest 30 day all-cause mortality. Similarly, sub-cluster 3F, which had the second highest number of isolates, harboured a higher than average number of antimicrobial resistance and virulence genes. Conversely, isolates in sub-cluster 1B had the lowest numbers of antimicrobial resistance and virulence genes and the lowest 30-day all-cause mortality.

Comparing sub-clusters, we observed an increase in the number of clonal isolates, namely ST1421 and ST796, in the two van operon dominant sub-clusters, 2C and 3F respectively. In contrast we observed a low clonality of isolates and the absence of the van operon in sub-cluster 1B. Other than a high prevalence of the van operon, we were not able to identify additional antimicrobial resistance or virulence genes that offered sub-clusters 2C and 3F an advantage over the other sub-clusters. A significant difference in the 30-day all-cause mortality was observed in the four predominant VREfm sub-clusters (2B, 2C, 3D and 3F) compared to sub-cluster 1B. The findings suggests the presence of van operons contribute to the success of the clone and the increased 30-day all-cause mortality observed.

In sub-clusters 2C and 3F, we observed a dominance of the vanA operon and vanB operon respectively. However, the dominance of a particular van operon was not observed throughout a cluster. From the phylogenetic tree of clusters 2 and 3, basal isolates in sub-clusters 2A and 3A predominantly harboured a mixture of van operon type. With the exception of sub-cluster 3B, when moving further from the root in clusters 2 and 3, a gradual and sequential increase in the prevalence of a particular van operons was observed suggesting an increasing affinity between isolates and the dominant van operon type for that cluster.

Over the three-year study period, we observed a decrease in the proportion of isolates harbouring the vanB operon but an increase in isolates harbouring the vanA operon. The result, coupled with the 2011 report identifying a 98.4% prevalence in vanB type VREfm[32], indicates a shift in trend towards vanA VREfm in Australia.

Although *E*. *faecium* isolates were collected from every region in Australia, limitations of this study include the difference in the number of participating laboratories and population demographics in each region. Additionally, frequent patient transfers between NT and SA, Tas and Vic, and the ACT and NSW, has resulted in major cross regional movement of patients. Therefore, in this study, we are unable to make accurate observations comparing the regional distribution of isolates across Australia. However, for most regions, we did observe specific region-dominant sub-clusters accounting for at least 45% of isolates. Additionally, we observed isolates from cluster 2C were collected in the fewest locations (NSW, Vic, SA and ACT) suggesting a more confined geographical distribution. A further limitation of the study was the clinical data of the patients was not available and therefore could not be analysed.

In our study, we observed several discrepancies between the relationship of STs described by the BURST diagram compared to SNP based molecular phylogeny. For example, isolates in sub-cluster 2C typed as ST1421 and ST1424 and isolates in sub-cluster 3F typed as ST203 and ST796 were observed to be distantly related by BURST but closely related by SNP phylogeny. SNP inferred isolate relationship offers a much higher resolution. As such, the use of BURST modelling may not be accurate in depicting relationships between *E*. *faecium* strains.

In conclusion, our study has shown *E*. *faecium* isolates from 2015 to 2017 causing BSI in Australia could be classified into three phylogenetic clusters. Moreover, each cluster can be characterized by a dominant van operon (or the lack of one) which we have shown to be key in the dissemination of isolates. Our findings also show, unlike in 2011 when VREfm in Australia was primarily due to the *vanB* operon, the presence of a highly successful *vanA* dominant strain identified in sub-cluster 2C have resulted in the co-dominance of vanA and vanB VREfm in Australia. Our analysis has highlighted clinically important strains of *E*. *faecium* in sub-cluster 2C and 3F which should be closely monitored in future surveillance. With the phylogeny of VREfm in Australia established in this study, future surveillance can now identify the introduction or emergence of new *E*. *faecium* strains with a much higher resolution compared to MLST. Additionally, with regular timely monitoring, individual hospitals can use the detection of clinically important VREfm strains, such as those identified in sub-cluster 2C and 3F as early warnings of potential outbreaks and therefore appropriate infection control procedures could be commenced earlier than previously. As *E*. *faecium* is an emerging nosocomial pathogen with extended antibiotic resistance, an online resource offering rapid typing and phylogenetic relatedness linked to antibiotic resistance genes and clinical data would be very useful [33]. Further studies of the van operon and genes associated with the operon will contribute to our understanding of the evolution of enterococci in the hospital environment and assist in the implementation of successful control strategies.

## Supporting information

S1 Table Percentage of predominant virulence factors identified in the 11 major *Enterococcus faecium* multilocus sequence types. (DOCX)

S2 Table Percentage of predominant resistance genes identified in the 11 major *Enterococcus faecium* multilocus sequence types. (DOCX)

S3 Table Hospitals and members participating in the AGAR AESOP from 2015–2017. (DOCX)

S1 Fig eBURST of the Australian Group on antimicrobial resistance Australian Enterococcal Sepsis Outcome Programs 2015–2017 *Enterococcus faecium* multilocus sequence types and isolates from the reference database (https://pubmlst.org). Lines between nodes indicate single locus variants, circle size indicates isolate numbers and yellow and blue circles indicate founders.
(TIFF)
