# Supplementary material for: A three-year whole genome sequencing perspective of Enterococcus faecium sepsis in Australia
Source: PLoS One. 2020 Feb 14;15(2):e0228781. doi: 10.1371/journal.pone.0228781 (PMC7021281; doi:10.1371/journal.pone.0228781)
Supplement: S1 Table — (DOCX) [file pone.0228781.s001.docx]

# Appendix

Supplementary Table 1. Percentage of predominant virulence factors identified in the 11 major *Enterococcus faecium* multilocus sequence types.

| ST | *acm* | *bopD* | *bsh* | *clpP* | *cpsF* | *ecbA* | *fss3* | *psaA* | *sgrA* |
| --- | --- | --- | --- | --- | --- | --- | --- | --- | --- |
| 17 | 100.0 | 99.1 | 100.0 | 100.0 | 0 | 75.2 | 75.2 | 76.9 | 93.2 |
| 18 | 100.0 | 100.0 | 100.0 | 100.0 | 0.0 | 5.0 | 10.0 | 5.0 | 70.0 |
| 78 | 100.0 | 97.6 | 100.0 | 100.0 | 21.4 | 0.0 | 95.2 | 95.2 | 52.4 |
| 80 | 100.0 | 100.0 | 99.0 | 100.0 | 0.0 | 1.9 | 46.6 | 44.7 | 97.1 |
| 192 | 100.0 | 100.0 | 100.0 | 100.0 | 0.0 | 95.2 | 100.0 | 100.0 | 95.2 |
| 203 | 100.0 | 100.0 | 100.0 | 100.0 | 0.0 | 79.4 | 88.9 | 95.2 | 95.2 |
| 262 | 100.0 | 100.0 | 100.0 | 100.0 | 0.0 | 0.0 | 19.0 | 19.0 | 9.5 |
| 555 | 98.9 | 100.0 | 100.0 | 100.0 | 0.0 | 0.0 | 100.0 | 96.7 | 94.5 |
| 796 | 99.4 | 100.0 | 100.0 | 100.0 | 0.0 | 1.9 | 100.0 | 96.9 | 96.2 |
| 1421 | 100.0 | 100.0 | 92.6 | 100.0 | 0.0 | 94.6 | 93.3 | 94.6 | 91.3 |
| 1424 | 100.0 | 100.0 | 97.2 | 100.0 | 0.0 | 95.8 | 91.5 | 95.8 | 97.2 |
