# Supplementary material for: A three-year whole genome sequencing perspective of Enterococcus faecium sepsis in Australia
Source: PLoS One. 2020 Feb 14;15(2):e0228781. doi: 10.1371/journal.pone.0228781 (PMC7021281; doi:10.1371/journal.pone.0228781)
Supplement: S2 Table — (DOCX) [file pone.0228781.s002.docx]

Supplementary Table 2. Percentage of predominant resistance genes identified in the 11 major *Enterococcus faecium* multilocus sequence types.

|  | Aminoglycoside | | | | | | Chloramphenicol | | Tetracycline | | | | MLSb^1^ | | | Vancomycin | | Trimethoprim | EMSb^2^ | Lincosamide |
| --- | --- | --- | --- | --- | --- | --- | --- | --- | --- | --- | --- | --- | --- | --- | --- | --- | --- | --- | --- | --- |
| ST | *aac(6')-aph(2'')* | *aadE* | *ant(6)-Ia* | *aph(2'')-Ie* | *aph(3')-III* | *spc* | *cat(pC221)* | *cat* | *tet(L)* | *tet(M)* | *tet(S)* | *tet(U)* | *erm(A)* | *erm(B)* | *erm(T)* | *vanA* | *vanB* | *dfrG* | *msr(C)* | *lnu(B)* |
| 17 | 11.1 | 0.0 | 27.4 | 5.1 | 70.1 | 1.7 | 0.0 | 0.0 | 19.7 | 33.3 | 0.0 | 55.6 | 1.7 | 79.5 | 12.0 | 1.7 | 12.0 | 29.1 | 100.0 | 0.0 |
| 18 | 25.0 | 20.0 | 5.0 | 0.0 | 25.0 | 0.0 | 0.0 | 25.0 | 85.0 | 25.0 | 0.0 | 0.0 | 0.0 | 55.0 | 30.0 | 0.0 | 20.0 | 85.0 | 100.0 | 25.0 |
| 78 | 19.0 | 0.0 | 9.5 | 21.4 | 31.0 | 0.0 | 0.0 | 0.0 | 66.7 | 23.8 | 0.0 | 38.1 | 0.0 | 40.5 | 35.7 | 2.4 | 88.1 | 71.4 | 100.0 | 2.4 |
| 80 | 3.9 | 1.0 | 31.1 | 0.0 | 36.9 | 0.0 | 1.0 | 0.0 | 62.1 | 12.6 | 26.2 | 25.2 | 0.0 | 46.6 | 35.0 | 32.0 | 14.6 | 68.9 | 100.0 | 3.9 |
| 192 | 0.0 | 0.0 | 71.4 | 0.0 | 100.0 | 0.0 | 0.0 | 0.0 | 14.3 | 4.8 | 0.0 | 4.8 | 0.0 | 100.0 | 0.0 | 0.0 | 0.0 | 0.0 | 100.0 | 0.0 |
| 203 | 15.9 | 0.0 | 74.6 | 1.6 | 77.8 | 0.0 | 11.1 | 0.0 | 1.6 | 82.5 | 0.0 | 28.6 | 0.0 | 95.2 | 0.0 | 14.3 | 49.2 | 1.6 | 100.0 | 30.2 |
| 262 | 0.0 | 4.8 | 0.0 | 0.0 | 0.0 | 0.0 | 0.0 | 61.9 | 85.7 | 4.8 | 0.0 | 9.5 | 0.0 | 85.7 | 0.0 | 0.0 | 0.0 | 90.5 | 100.0 | 4.8 |
| 555 | 25.6 | 0.0 | 42.2 | 0.0 | 45.6 | 1.1 | 0.0 | 0.0 | 2.2 | 53.3 | 0.0 | 4.4 | 1.1 | 90.0 | 1.1 | 0.0 | 47.8 | 93.3 | 100.0 | 37.8 |
| 796 | 38.4 | 0.0 | 3.8 | 0.0 | 3.8 | 1.9 | 0.6 | 0.6 | 0.0 | 95.6 | 0.6 | 30.2 | 1.9 | 90.6 | 0.0 | 0.6 | 95.0 | 97.5 | 100.0 | 1.3 |
| 1421 | 8.7 | 0.0 | 0.0 | 0.0 | 91.3 | 97.3 | 0.0 | 0.0 | 10.1 | 6.7 | 0.0 | 34.9 | 94.6 | 96.6 | 2.7 | 74.5 | 0.7 | 92.6 | 99.3 | 0.0 |
| 1424 | 4.2 | 0.0 | 0.0 | 0.0 | 69.0 | 94.4 | 0.0 | 0.0 | 22.5 | 2.8 | 2.8 | 29.6 | 93.0 | 91.5 | 0.0 | 60.6 | 1.4 | 97.2 | 100.0 | 0.0 |

^1^ Macrolide, Lincosamide and Streptogramin B (MLSb)

^2^ Erythromycin, Macrolide and Streptogramin B (EMSb)
