# Supplementary material for: A three-year whole genome sequencing perspective of Enterococcus faecium sepsis in Australia
Source: PLoS One. 2020 Feb 14;15(2):e0228781. doi: 10.1371/journal.pone.0228781 (PMC7021281; doi:10.1371/journal.pone.0228781)
Supplement: S3 Table — (DOCX) [file pone.0228781.s003.docx]

Supplementary Table 3. Hospitals and members participating in the AGAR AESOP from 2015-2017.

| Hospital | Participant |
| --- | --- |
| Alfed Hospital | Denis Spelman  Amanda Dennison  Rose Bernhard |
| Alice Springs Hospital | James McLeod |
| Austin Hospital | Peter Ward  Elizabeth Grabsch  Paul Johnson |
| Cairns Base Hospital | Bronwyn Thoett |
| Concord Repatriation General Hospital | Tom Gottlieb  Graham Robertson |
| Fiona Stanley Hospital | David McGechie |
| Flinders Medical Centre | Kelly Papanaoum  Nicholas Wells  Xiao Chen |
| Gold Coast Hospital | Petra Derrington  Sharon Dal-Cin  Sam Maloney  Brian Gorman |
| Greenslopes Hospital, Sullivan Nicolaides Pathology | Jenny Robson  Georgia Peachey |
| John Hunter Hospital | Rodney Givney  Ian Winney |
| Joondalup Hospital | Shalinie Perera  Ian Meyer |
| Lady Cilento Childrens Hospital | Clare Nourse  Narelle George |
| Launceston Hospital | Pankaja Kalukottege  Kathy Wilcox |
| Monash Medical Centre and Monash Childrens Hospital | Tony Korman  Despina Kotsanas |
| Nepean Hospital | James Branley  Donna Barbaro  Linda Douglass |
| North -West Regional Western Australia | Michael Leung |
| Perth Children's Hospital | Chris Blyth |
| Prince Charles Hospital | Robert Horvath |
| Princess Alexandra Hospital | Naomi Runnegar  Joel Douglas |
| Royal Adelaide Hospital and The Women and Children's Hospital | Morgyn Warner  Kija Smith |
| Royal Brisbane And Women's Hospital | Graeme Nimmo  Narelle George |
| Royal Children’s Hospital | Andrew Daley  Gena Gonis |
| Royal Darwin Hospital | Rob Baird  Jann Hennessy |
| Royal Hobart Hospital | Louise Cooley  David Jones |
| Royal North Shore Hospital | George Kotsiou  Peter Huntington |
| Royal Perth Hospital | Owen Robinson |
| Royal Prince Alfred Hospital | Sebastian van Hal  Bradley Watson  Alicia Beukers |
| Sir Charles Gairdner Hospital | Ronan Murray  Jacinta Bowman |
| St John of God Pathology, Murdoch Hospital | Fay Kappler  Sudha Pottumarthy-Boddu |
| St Vincent's Hospital, Melbourne | Mary Jo Waters  Lisa Brenton |
| St Vincents Hospital, Sydney | Jock Harkness  David Lorenz |
| The Canberra Hospital | Peter Collignon.  Susan Bradbury |
| Westmead Hospital | Andrew Ginn |
| Wollongong Hospital | Peter Newton  Melissa Hoddle |
