# Supplementary figures and images for: A three-year whole genome sequencing perspective of Enterococcus faecium sepsis in Australia
Source: PLoS One. 2020 Feb 14;15(2):e0228781. doi: 10.1371/journal.pone.0228781 (PMC7021281; doi:10.1371/journal.pone.0228781)

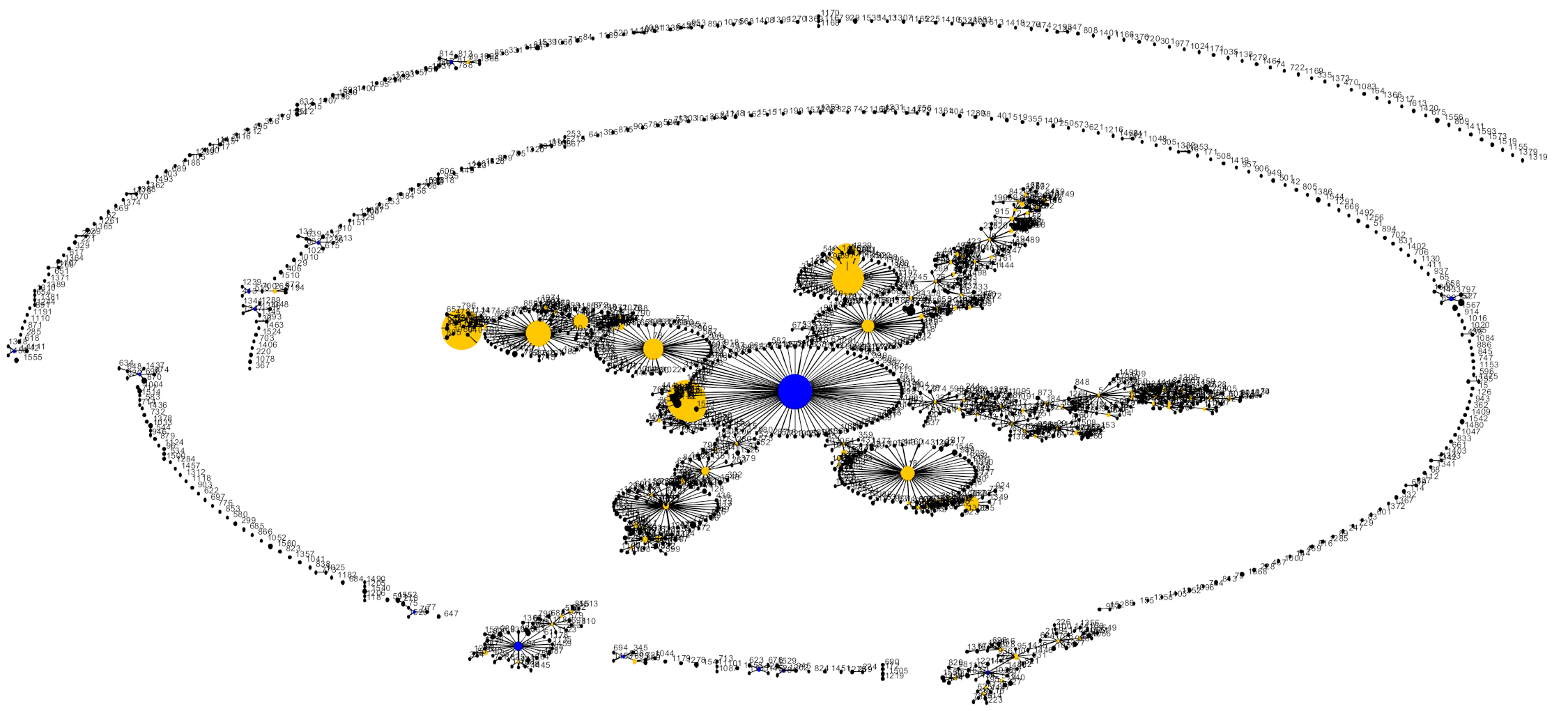

Supplement: S1 Fig — Lines between nodes indicate single locus variants, circle size indicates isolate numbers and yellow and blue circles indicate founders. (TIFF) [file pone.0228781.s004.tiff]
